# Is it only the regulatory status? Broadening the debate on cisgenic plants

**DOI:** 10.1186/s12302-017-0120-2

**Published:** 2017-06-26

**Authors:** Lilian van Hove, Frøydis Gillund

**Affiliations:** Society, Ecology and Ethics Department, GenØk Centre for Biosafety, SIVA Innovation Centre, 9294 Tromsø, Norway

**Keywords:** Cisgenic plants, Late blight resistant potato, Broader risk assessment, Stakeholder deliberation, Responsible governance of agriculture

## Abstract

In current debates on emerging technologies for plant breeding in Europe, much attention has been given to the regulatory status of these techniques and their public acceptance. At present, both genetically modified plants with cisgenic approaches—using genes from crossable species—as well as transgenic approaches—using genes from different species—fall under GMO regulation in the EU and both are mandatorily labelled as GMOs. Researchers involved in the early development of cisgenic GM plants convey the message that the potential use and acceptance of cisgenic approaches will be seriously hindered if GMO regulations are not adjusted. Although the similar treatment and labelling of transgenic and cisgenic plants may be a legitimate concern for the marketability of a cisgenic GM plant, there are concerns around their commercialization that reach beyond the current focus on (de)regulation. In this paper, we will use the development of the cisgenic GM potato that aims to overcome ‘late blight’—the most devastating potato disease worldwide—as a case to argue that it is important to recognize, reflect and respond to broader concerns than the dominant focus on the regulatory ‘burden’ and consumer acceptance. Based on insights we gained from discussing this case with diverse stakeholders within the agricultural sector and potato production in Norway during a series of workshops, we elaborate on additional issues such as the (technical) solution offered; different understandings of the late blight problem; the durability of the potato plant resistance; and patenting and ownership. Hence, this paper contributes to empirical knowledge on stakeholder perspectives on emerging plant breeding technologies, underscoring the importance to broaden the scope of the debate on the opportunities and challenges of agricultural biotechnologies, such as cisgenic GM plants. The paper offers policy-relevant input to ongoing efforts to broaden the scope of risk assessments of agricultural biotechnologies. We aim to contribute to the recognition of the complex socio-ecological, legal and political dimensions in which these technological developments are entangled as a means to acknowledge, discuss and respond to these concerns and thereby contribute to more comprehensive and responsible developments within agricultural biotechnology.

## Background

The regulatory status and public acceptance of emerging technologies for plant breeding have been considerably debated among scholars, policymakers, innovators and non-profit organizations, though there has been little public debate on the topic [1, 2]. Recent developments within biotechnology, such as cisgenic approaches—the genetic modification (GM) of a recipient organism with genes from a crossable, sexually compatible, variety of the same or closely related species [3]—bring to the fore the debate on the applicability of current genetically modified organisms (GMO) regulation and whether exemptions should be made for new plant breeding techniques [4–9]. Cisgenic GM potato that is resistant to ‘late blight’ is one example of a product developed using emerging plant breeding technologies, and serves as a good illustration of the complex socio-ecological, legal and political dimensions in which these agricultural biotechnologies are entangled. As to date, potato production is significantly plagued by late blight which is the most devastating potato disease worldwide. A fungus-like organism called *Phytophthora infestans* causes the disease. It is estimated to result in an annual global loss of 7 billion Euros [10] and present control measures in conventional potato production involve excessive use of fungicides. Improving host plant resistance, and thereby reducing the need for fungicides, is considered to be the most sustainable way to control late blight [11, 12]. Traditional introgression breeding for late blight resistance is challenging, mainly because the potatoes are genetically complex and reproduce vegetatively. New plant breeding techniques such as cisgenic approaches hold promises. It is possible to use the cisgenic approach to improve late blight resistance in potato cultivars because the genes that make potatoes resistant to late blight—so-called resistance (R) genes—originate from wild potato species found in South and Central America [13]. Therefore, in recent years, the use of genetic modification to develop a late blight resistant potato has shifted from a transgenic approach—a genetic modification of a recipient organism with genes from an *unrelated* organism—to a cisgenic approach. This shift is driven by the potential that if successful, cisgenic plants will be more accepted by public as the inserted gene is from crossable species and the product is thereby more similar to traditionally bred plants.

In the debate about GM regulation in Europe and specifically the difference between ‘cisgenic’ and ‘transgenic’ approaches, it is argued that exempting cisgenic plants from GMO regulations may herald a new future for agricultural biotechnology [14]. Currently, no distinction between cisgenic and transgenic approaches is made within European GMO regulation and this has become a highly controversial issue. Proponents of deregulation argue that a cisgenic plant can have the exact same properties and is as safe as a plants obtained by traditional introgression breeding [7, 15–17]. It is therefore considered inconsistent that plants with the same properties are differently regulated. Furthermore, it is questioned whether cisgenic plants fall under the definition of a GMO, as this definition states that a “GMO means an organism […] in which the genetic material has been altered in a way that does not occur naturally by mating and/or natural recombination” [18]. As cisgenic potatoes do not have a novel combination of genetic material compared to conventionally bred plants, it is argued that they should therefore not fall under the GMO legislation [15]. In most jurisdictions, for example in the Cartagena Protocol on Biosafety and the European GMO legislation, there are both process- and product-related criteria, though the focus in the EU has been on process criteria (e.g. [9]). Those in favour of a strict regulation indeed point to the process criteria to argue that the same technique is applied with cisgenic and transgenic approaches and that therefore cisgenic should fall under the GMO regulation. Another argument stresses that one of the reasons for current regulation of GMOs is the traceability and labelling of the GMO products so as to guarantee consumers’ right to know which products contain GMO. If cisgenics is exempted from GMO regulation, consumers would not be able to actively choose products that they know are not genetically modified [14].

The outcome of the re-evaluation of the EU GMO legislation was expected by the end of 2015, but the process keeps delayed and currently no decision has been made. Indeed, the re-evaluation of the GMO legislations and the request for exempting cisgenic approaches from this regulation arguably open up sensitive issues about GMO regulation. For example, whether all GMOs should be regulated in the same way, whether new biotechnologies in plant breeding techniques should fall under the GMO definition, or whether the regulation of GMOs should be process or product based [7].

Researchers involved in the development of cisgenic GM ‘late blight resistant’ (LBR) potato actively engage in these policy discussions, conveying a message that burdensome GMO regulations prevent the commercialization of cisgenic plants and that their future use and acceptance will be seriously hindered if GMO regulations fail to differentiate between cisgenic and transgenic organisms [8, 10, 15, 17, 19–21]. Indeed, the need for a re-evaluation of the current GMO regulation is also wanted because of new genome editing techniques, such as CRISPR/Cas9 and synthetic biology that were not existing when the current regulation was established. The rise of these techniques, such as CRISPR/Cas9—which is currently considered as the most precise and site-specific genome editing technique and is used to delete, insert or replace gene sequences [22, 23]—are described as promising developments for improving crop traits and increasing the use of plant genetic resources in pre-breeding and breeding work [24, 25]. Combining cisgenic approaches with new genome editing techniques could enable more efficient use of genetic resources and possibly more precise crop breeding. For instance, using CRISPR/cas9 to introduce cisgenes into a plant could potentially build trust in the precision and safety of cisgenic approaches and thereby “tackle some objections to the application of biotechnologies in agriculture” [24]. Furthermore, synthetic biology approaches have the potential for designing disease resistance in crops and could enable the construction of novel, resilient immune response networks in plants [26]. A synthetic biology approach could potentially be used to construct synthetic R genes or multi-R-gene constructs to improve late blight resistance. This could complement current potato breeding approaches that exploit naturally occurring R genes found in wild potato species.

Irrespective of the potential opportunities that new biotechnological approaches such as CRISPR/Cas9, cisgenic or synthetic biology could bring about, GMOs currently remain controversial. When it comes to cisgenic plants, there are other concerns that are hindering a successful commercialization than the current focus on the (de)regulation. These concerns include issues such as corporate control over seed markets and the food chain, consumers’ and farmers’ right to know and choose, the coexistence of different agricultural production systems, intellectual property rights, power relations, scientific uncertainties, ambiguities and values underlying the knowledge development and concerns about the human domination of nature [27–29]. The persistent controversies in views and debates on GMOs in general contain polarized arguments that sustain permanent different viewpoints and disagreement about the facts and values and their interwoven nature [30]. These controversies that surround agricultural biotechnologies and the aim to develop truly “responsible governance of agricultural biotechnology” [29] give little reason to assume that clarifying the regulatory status of cisgenic plants and exempting them from GMO regulations are sufficient to assure their marketability and/or public acceptance towards this technology. Moreover, new European legislation on GMOs allows Member States to restrict the cultivation of GMOs on their own territory based on socio-economic impacts, environmental or agricultural policy objectives, or with the aim to avoid the unintended presence of GMOs in other products (Directive 2015/412). This reflects the increased international recognition and significance to consider a broader range of issues beyond the environmental and human safety-related aspects—such as sustainability, social utility and ethical justifiability—when assessing the use of agricultural biotechnologies [31, 32]. Furthermore, the recognition to consider a broader assessment frame has been accompanied with a shift in the relationship between science and society, going from ‘science and society’ to ‘science in and with society’ (e.g. [33, 34]), or ‘science with, and for society’ (European funding program Horizon 2020). This has resulted in an increase of attention on addressing societal needs, anticipating impacts, assessing alternatives, and conducting public outreach of scientific work. These approaches aim for more transparency in research processes and outcomes, and rely on a participatory and inclusive process (e.g. [35–37]). This shift has changed how research, research-based policies, technological developments and technology assessments are developed and performed.

Motivated by these thoughts, and particularly the call for broadening risk assessments of GMOs through participatory processes, we organized a series of three workshops with the aim to identify stakeholder perspectives on biotechnological developments within agriculture—such as cisgenic LBR GM potato. In these workshops, stakeholders involved in the potato production and the agriculture sector in Norway discussed concerns and questions related to sustainability, ethical and social considerations raised by the potential cultivation of cisgenic LBR GM potato in Norway. Two workshops focused on sustainability aspects and used the Problem Formulation Option Assessment method as a way to stimulate discussion (see [38] for more details on the use of this method and specific outcomes). The third workshop focused on social and ethical aspects and used the Ethical Matrix as a starting point for discussion (see [39] for more details on this workshop and the use of the Ethical Matrix during their workshop). An interesting data set was produced from the three workshops which when analysed thematically highlight a number of important issues raised by the stakeholders, namely: the (technical) solution offered; the problem phrasing of the potato disease; the durability of the resistance of the GM potato; and patenting and ownership. By drawing on questions, opinions, concerns and uncertainties that this diverse group of stakeholders shared or raised on these issues, we argue for a broader recognition of, and reflection on the implications arising from the cultivation of cisgenic plants, and cisgenic LBR potato within Europe in particular. The stakeholders participating included potato breeders, seed potato retailers, seed potato and potato producers, representatives from the potato processing industry, agricultural advisors, regulators and representatives of environmental, agricultural and consumer interests and researchers within the fields of crop plants and plant pathogens, and social, economic and ethical issues related to GMOs. The main intention with these stakeholder discussions was to explore the diversity of views among the participants in the workshops, rather than reaching any form of consensus or arrive at representative conclusions. Consequently, the participants did not always agree with each other about arguments, questions, concerns and claims made during the discussions and we recognize that many more concerns and questions than we address can be identified and would benefit from further research and reflection.

In this paper, we highlight the issues that were given particular attention during the workshop discussions by stakeholders. The paper is structured in four sections in which each of the issues (i) the (technical) solution offered; (ii) the problem phrasing of the potato disease; (iii) the durability of the resistance of the GM potato; (iv) and patenting and ownership are described in more detail. We describe how the issues were addressed by workshop participants, present relevant scientific literature in the field and make suggestions for future research needs. A key contribution of this paper is the empirical insight on stakeholder perspectives on emerging plant-breeding techniques, thereby underscoring the importance of broadening the scope of the debate on the opportunities and challenges of agricultural biotechnologies, such as cisgenic GM plants. In terms of its policy relevance, the paper offers a valuable contribution to ongoing efforts and a growing need to broaden the scope of risk assessments of agricultural biotechnologies, as for instant reflected in the new European legislation on GMOs (Directive 2015/412). By addressing these concerns, we aim to broaden the debate so as to acknowledge, discuss and contribute to respond to them.

## A technical fix to consumer concerns

The shift from transgenic to cisgenic approaches in the development of LBR GM potato was based on the availability of resistance (*R*) genes in wild potato species. The assumption was that this approach would be ethically more acceptable to the public as these genes are from crossable species instead of different species [21]. However, past and present debates on this topic, and the difficulty to decide on regulation for it, have shown that there are additional concerns that reach beyond the regulatory questions. Indeed, when the development of cisgenic technologies is considered to be “*a result of taking the opinions and concerns of consumers seriously*” [40] and is aimed to respond to consumer concerns, it fails to recognize the broader issues that are persistently addressed within society about GM approaches in general. During the workshops, it became clear that most of the Norwegian stakeholders in the potato and agriculture sector consider it as a reasonable assumption that cisgenic plants will decrease consumer concerns to some degree. Still, they highlighted that it is too simplistic to think that this technical fix is sufficient to alleviate all concerns associated with GM crops.

Moreover, there is no scientific consensus on the safety of cisgenic plants. For example, the GMO panel of the European Food and Safety Authority [41] concluded in 2012 that the risks associated with cisgenic and traditionally bred plants are the same, while transgenic plants may result in novel risks. This conclusion was gene based, meaning that because the genes are known to be safe, the cisgenic approach was also considered safe. However, even if the inserted gene is known, this does not mean that uncertainties associated with the transformation process are known. Hence, in contrast to the conclusion of the EFSA, it is argued that, in terms of food and environmental safety, cisgenic approaches cannot be regarded as equivalent to conventional breeding. Neither can the safety of the products resulting from traditional breeding and cisgenic technologies be considered as similar, based on the unknown behaviour and effects of the genes in the transformation process and their functioning within the mechanisms of the host plant [42–44]. Furthermore, the issues reaching beyond the safety of the cisgenic plant seem to get little attention from researchers in the field of these emerging breeding techniques. For them, it seems that the ability of cisgenic plants to get more public acceptance is a common ground to pursue cisgenic approaches and advocate for a less strict regulation [2]. Although the ‘more natural’ approach of cisgenic compared to transgenic may affect the view of consumers to this technique to some degree, cisgenic breeding remains a genetic modification, which remains controversial, and consumers still favour, for example, labelling of such a technique [14].

The assumption that cisgenic plants will alleviate the reluctant attitude of consumers raised concerns among some of the Norwegian stakeholders about the effect this may have on the consumers’ trust in their farming practice, their products, and the agriculture sector in general. This prevented these stakeholders to fully embrace this technological shift as the solution for late blight. Their concerns go beyond ambiguities on the regulatory status of cisgenic plants or safety issues, to include their relationship with consumers and the potential damage that a lack of transparency, communication and research of alternatives may cause. Moreover, some stakeholders were concerned that a loss of trust from the public could result in a decrease of subsidies for the agriculture sector. For years, the Norwegian agriculture sector receives one of the highest governmental support levels among the OECD countries [45, 46]. Some of the stakeholders emphasized that the current financial support from the government stems from the trust in quality of domestic agricultural products. These subsidies constitute a large share of the income of Norwegian farmers and thus an important reason for them to carefully assess the potential implications that different methods for plant breeding may have on their trustworthiness.

The limited amount of empirical research on consumer’s acceptance of GM food indicates different perceptions of consumers towards cisgenic and transgenic plants; this arguably demonstrates that the acceptance of cisgenic approaches is not straightforward. A study in 2010 demonstrates a more reluctant than tolerant attitude towards GM food in Europe [47], as does a study in 2017 under Norwegian consumers [48]. Research by Lusk and Rozan [49] indicates a higher acceptance of cisgenic than transgenic approaches for GM food, based on nationwide surveys in the US and France. Their research demonstrates a significant difference between these nations, with a twice as high acceptance level in the US. Moreover, European citizens perceive a lack of knowledge on GM approaches, hindering them to make a well-informed decision about their willingness to consume GM products [50]. Although Delwaide et al. claim that the different perceptions on transgenic and cisgenic plants confirm the hypothesis that public will be more tolerant to the latter than the former, it is not yet clear what the conditions for acceptance of a GM application are. Indeed, a research performed by Mielby et al. [51] indicates that more knowledge of different GM approaches does not lead to consumers developing an attitude based on differences between methods (i.e. transgenic or cisgenic). Rather, consumers consider the purposes of GM applications as important, such as a medical application or food application. Furthermore, consumers choose to depend on people they consider experts and trustworthy to make informed decisions. This gives trust an important role in the view people develop of GM food [52–54].

Overall, there is relatively limited information available about current perception of European public on cisgenic and transgenic plants to get a comprehensive and accurate representation of consumers’ preference and public acceptance. More empirical studies and surveys are required to reveal and document the support base from public, and assess whether a further development of cisgenic plants is safer, desirable, acceptable and can be marketable. Additionally, the debates on cisgenic approaches would benefit from including broader issues mentioned by different stakeholders within the potato and agricultural industry—both on the level of cisgenic approaches as well as the more general level of GMOs. Failing to acknowledge these broader issues and relying on a technical fix to overcome the safety and consumer concerns with cisgenic GM approaches potentially sustains a scattered and under-communicated discussion and severely complicates constructive and responsible developments within agricultural biotechnology.

## The importance to recognize alternatives

Different understandings of the late blight problem, approaches to breed for more resistant potato varieties and “models for agriculture” will lead to a different set of farm practices to try to fight late blight. In this particular case, characterizing the problem, approaches and solutions is mostly framed within the currently dominant approach of conventional potato production that involves excessive use of fungicides to control the disease [3, 10]. The message conveyed is that despite considerable efforts during the last 150 years, traditional introgression breeding for durable late blight resistance has largely been unsuccessful [13]. Genetic modification is expected to contribute in solving these challenges as it allow researchers to introduce several *R* genes into a potato variety in which the qualities appreciated by farmers, processors and consumers remain the same.

Reflection on the dominant approach in research and governance of agriculture reveals the different values on which the approach is based, and may lead to different practices, evaluations and perspectives on cisgenic plants [55]. Risk assessment of GM crops is however, typically narrowly framed, where technological benefits and risks are compared to current dominating production systems (most commonly conventional agriculture). This may cause a tunnel vision, where only particular problems, tactics and solutions are discussed. Contrary to this approach, we argue that it is crucial to critically reflect on whether the solution that is offered is responding to the societal need that it is supposed to address. Moreover, the true origins of the problem should be identified, different future visions explored and alternative approaches and solutions investigated.^1^ Exploring alternative approaches includes considerations of their benefits, adverse effects, uncertainties, ambiguity, existing knowledge gaps and the changes required for large-scale implementation of this alternative [56].

During our workshops, some stakeholders emphasized that the message conceived seems to be that, based on the current (dominant) agricultural model, improving host plant resistance is the best strategy to fight late blight, and that GM is the only means to develop potato varieties with durable resistance to late blight that also satisfy consumer demands. They stressed that there is limited attention and investigation of alternative approaches to fight late blight. Stimulating critical reflection is useful to reveal the understanding of a particular societal need, problem, and proposed solution by different stakeholders or different agricultural models. It is also a useful means to demonstrate the different interests and future visions of stakeholders (e.g. what kind of society do we want?) and opens up opportunities to direct technological developments in ways that are perceived to be socially desirable [34]. A way to manifest critical reflection on the underlying values of a technological development or an agricultural practice is to open up the assessment processes to a more participatory model, with deliberation among different stakeholders. Indeed, the current trend to emphasize the importance of recognizing underlying values, assumption and beliefs to achieve “responsible governance of agricultural biotechnology” [29] underscores the significance to critically reflect on these hidden values, norms, assumption and visions within agriculture to understand the different perspectives and solutions offered.

The aspiration for more critical reflection was also shared by some of the stakeholders, who highlighted that there is little consideration of more overarching questions related to the late blight problem and challenged the “narrow scope” in the frames of conventional large-scale potato production. For example with regard to whether the severity of the late blight problem is partly a result of industrial agriculture dominated by large-scale monoculture production. These stakeholders called for a broader problem formulation frame, pointing to questions related to the role of agricultural policies and regulations in promoting large-scale industrial potato production and “roads not taken” when searching for alternative approaches to breed for late blight resistance.

Current agricultural policy, incentives, structures and regulation are implicitly focussed and structured to support and sustain conventional agriculture as the dominant approach, for example by defining ‘organic agriculture’ as a separate category whereas conventional agriculture is meant with agriculture (e.g. [57, 58]). Critically reflecting on the effect of this dominant approach, acknowledging the complex socio-ecological systems and networks of which agricultural biotechnologies are part of is important as well as being open to learn from other agriculture systems [e.g. 28]. Learning from other agricultural systems could also facilitate responding to broader concerns, such as the contribution to sustainable development, the societal needs and the ethical justifiability of a technological development within agriculture (As an example, see http.//www.agriculturesproject.org). An assessment of a biotechnological application that takes these aspects into account benefits from input and deliberation with for example practitioners of agriculture and those working with the governance of agriculture, rather than solely expert-driven or academic actors focusing on agriculture. This may lead to create more understanding between different stakeholder groups, enables responsible approaches to generate more support for decisions and responds to the opinions and concerns of stakeholders in a desirable way.

## The quest for durable late blight resistance

A host plant and its pathogen (e.g. virus, bacteria or fungus) are typically in a constant evolutionary arms race. In our case, the potato plant is continuously trying to develop ways to defend itself from late blight, while *P. infestans* is continuously searching for ways to break these defence mechanisms. *P. infestans* has a remarkable capacity to rapidly adapt to resistant host plants [59], and *R* genes introduced to commercial potato varieties through traditional introgression breeding have consequently been defeated shortly after the potato variety are put in commercial production [13]. Traditional breeding for potatoes with late blight resistance that lasts over time has therefore been a vexing challenge.

One of the main aims for using genetic modification when developing LBR potato varieties is to achieve resistance that is lasting over time [10]. Novel molecular technologies have advanced researchers' ability to identify and characterize genes that provide late blight resistance and transfer these genes from wild potato species to commercial ones [13]. Still, several questions remain about the molecular mechanisms underlying the observed resistance of potato plants [60–62]. Similarly, more research is needed to advance the understanding of the molecular and genetic mechanisms of virulence and adaptability in the *P. infestans* population [63]. Further research of these knowledge gaps is crucial in the search for ways to achieve durable LBR potato varieties. Still, these uncertainties are seldom acknowledged and openly discussed in public, as for instance illustrated with the lack of attention that these issue are given in a recent report written by leading European researchers involved in the development of cisgenic LBR potato [64]. Rather, the message conveyed by researcher working in this field is that GM potato will significantly contribute to a more sustainable potato production by providing the potato plant with a defence system that lasts over time [10, 20, 64–67]. They argue that this approach makes it easier for researchers to introduce several *R* genes into the same potato variety, which is expected to tip the ‘evolutionary’ balance in favour of the potato plant and against *P. infestans*, as it will be more difficult and time consuming for *P. infestans* to overcome multiple defence mechanisms at once. When this approach is presented to public, it is however largely under communicated that the durability of the cisgenic LBR GM potato can only be assessed in retrospect, meaning that it is problematic to predict how long the resistance will last until the potato is put in large-scale production and cultivated over time.

It is also important to be aware that successful—i.e. durable—application of the cisgenic LBR GM potato hinges on careful monitoring and development of resistant management strategies. This implies that the *P. infestans* population is monitored to detect if any of the *R* genes of a certain GM potato variety are broken by the pathogen, and that strategies to halt virulence development in the *P. infestans* population are established prior to large-scale cultivation [68]. These strategies must be adapted to the area where the GM potato will be grown. In Norway, for instance, the genetic diversity of the *P. infestans* population is particularly high [69], which may strengthen the adaptive potential of the pathogen, and possibly influences its ability to overcome resistance. Finally, it is important to clarify questions about the distribution of the responsibilities and the costs for carrying out monitoring and resistance management. Farmers and agricultural advisors must be sufficiently trained to carry out these tasks, which might be challenging, particularly in developing countries. For instance, one of the first outbreaks of resistance development among target organisms on GM Bt maize were reported in South Africa, partly explained by farmers’ failure to comply with resistant management strategies such as refuge requirements [70].

Some of the stakeholders questioned the durability of the GM approach, and emphasized that farmers and consumers may be left with the misconception that this approach is *the solution* to the late blight problem. As phrased by Mullins [71]: *“Demonstrating durability of generated resistance is key to underpinning grower confidenc*e”. Hence, a lack of transparency about the uncertainties associated with such a statement, as well as the conditions that this approach hinges on might result in a backlash against trust that farmers and public place on the researchers driving this technology. It may also limit the ability to open up for a broader and more inclusive discussion about how to address the uncertainties and limitations of this proposed solution, as these uncertainties and limitations are to a large extent unknown. Therefore, candidness and transparency about the uncertainties, knowledge gaps and limitations of using GM approaches to develop durable LBR potato varieties should be stimulated throughout the different levels of research and development of cisgenic plants.

## Patent rights and ownership

A major issue in the debate about GM technology is the intellectual property rights for plant varieties. The genetic modification of organisms is currently a patentable practice. However, patenting plant traits has given reasons for concerns by a broad range of the Norwegian stakeholders. Within the development of cisgenic crops, proponents emphasize that because the inserted gene stems from a crossable and/or sexually compatible variety; the obtained crop is in theory not different from a potentially traditionally bred crop. Thus for them, the similarity between cisgenic and conventional crops makes the product of cisgenic approaches closer to traditionally bred plant varieties, thereby arguing for easing the regulation of cisgenic crops. Despite this argued similarity, cisgenic crops are considered to be sufficiently different to be patentable. Although patent laws and requirements differ among nations, in general patents are granted if inventions are “new, involve an inventive step and are susceptible of industrial application” (Article 52, EPC 2016). What can be patented when it concerns biological inventions is the ‘biological material’—in this case the plant—possessing specific trait(s) as a result of the invention, or a process that enables a plant to be produced possessing specific trait(s) as a result of the invention (Article 8, DIRECTIVE 98/44/EC), for example the trait to be resistant to late blight. Nevertheless, if a biotechnological product fulfils these criteria and can obtain a patent, one could argue that it is reasonable to have an elaborated regulatory (safety) assessment in place to assess the risks, need and desire of this novel trait in the crop before it is released on the market. Therefore, a paradox emerges in the arguments in favour of deregulation of cisgenic crops, namely that the claim for novelty that is required to obtain a patent is the same reason why deregulation or easing the regulation of this method seems questionable, and arguably irresponsible. Indeed, if it is questioned whether cisgenic plants should fall under the (strict) GMO regulation, it simultaneously raises questions whether it still fulfils the criteria to be patentable. This illustrates that this discussion is not only about whether an elaborated safety assessment should be in place. It also touches upon the perceived ambiguity whether cisgenic approaches should be a patentable practice, thereby questioning the criteria of the ‘inventive step’.

The idea of a form of intellectual property right in agriculture is not new. In fact, developers of a new variety can get a “plant breeders’ rights” (PBR), giving the breeder the exclusive control over the breeding of a particular variety.^2^ The most concerning difference for stakeholders between PBR and patents however concerns the reuse of farm-saved seeds/potatoes to be used as seeds the next year. Under PBR, a potato farmer can save a part of its harvest to reuse the next year, which is significant in the Norwegian potato industry, as farmers take around 70–75% of their seed potatoes from their own crop.^3^ In contrast to a PBR, a patent on a plant trait does not allow the use of farm-saved seeds/potatoes and so farmers need to buy new seed potatoes every year, thereby increasing their costs significantly. Moreover, the difference between patents and PBR also affects breeding programmes. In general, the access to technology as well as genetic material is essential for the development of new plant varieties. Within PBR, this is regulated in the ‘breeder’s exemption’ that allows breeders to use each other’s varieties in breeding programmes without necessarily obtaining a licence or agreeing on financial compensation [72]. The importance of facilitating the development of new plant varieties is reflected in international treaties such as the International Union for the Protection of New Varieties of Plants (UPOV 1961/1978/1991), the Agreement on Trade-Related Aspects of Intellectual Property Rights (WTO–TRIPS—1994), and the International Treaty on Plant Genetic Resources for Food and Agriculture (IT PGRFA—2001). In contrast to PBR, patents always demand permission from the patent holder in order to be used in breeding programmes, leading to a longer procedure and potentially more costs.

Another concern related to the patentability of cisgenic crops concerns the traceability of cisgenic plants. This becomes extra complicated when traditional breeding becomes capable of developing and commercializing a variety with for example resistance to late blight, and both LBR potato via traditional breeding as well as GM co-exist. It then becomes difficult, if not impossible, to check whether a plant is cisgenic in retrospect and to keep control of where the patentable plant ends up, whether it crosses with other species and the assessment thereof. Moreover, there is uncertainty about the legal and economic consequences for farmers for unintended contamination through gene flow, as well as for the patent holder to potentially argue that his patent was breached.

For some of the Norwegian stakeholders, the concern with patenting is predominantly about the power and potential monopoly of biotech companies that obtain patents over plant varieties and the influence this can have on the seed and food prices, the power relations within the sector and the independent research on the safety of cisgenic plants and further uses, such as breeding by other research institutions (see also [73]). Entry of the patent system into for example the potato sector, contributes to the concentration and reduction of the diversity of companies and the possibilities for strategic use, which may lead to lack of clarity in the market and to monopolistic behaviour [74]. Patenting has the potential to increase the control of a few corporations over seeds and crops, thereby increasing farmer dependency and a reduction of biodiversity [27, 75–77]. The potential effects and implications for stakeholders and the uncertainty that patenting involves creates uneasiness among Norwegian stakeholders in the sector. Even though experimenting with patent regimes is ongoing, it seems that the fundamental principle of patenting is receiving significant resistance.

As cisgenic crops are considered GM products, they have to get through regulatory processes to get approval. Currently the costs for complying with regulatory processes can cost millions of euros for each application and typically takes years to complete [78, 79]. This burden of high costs to comply with regulatory processes is used as an argument in favour of exempting cisgenic plants from GM regulation, so that small and medium-sized enterprises can compete with the bigger, usually multinational corporations that are currently dominating the market (e.g. [8, 71, 80]). Therefore, in theory, easing regulation could benefit small enterprises. In practice however, it is questionable whether this will actually benefit small- and medium-sized enterprises, as they may easily be overruled by larger companies due to the larger capacity of these companies to develop new GM varieties. As patenting generates revenues for more research and enables seeking for the costly approvals of other inventions, it can cause a “Matthew effect” [81] in which the larger firms and institutes will get larger, while the small firms and research institutes are unable to keep up with this development and grow ever smaller.

Thus, the potential to patent cisgenic crops raises significant concerns about the implications for different actors in the sector and demands reflection on what kind of consequences patenting entails and calls for further investigation on the role of (different) patent policies within agriculture. Transparency about who will benefit, how power relations may shift or increase, and deliberation on these implications is warranted.

## Conclusion

In this paper, we have discussed important concerns in the debate about the development of LBR GM potato in Europe, which go beyond the regulatory status of cisgenic LBR potato and consumer acceptance of cisgenic plants. Although we recognize the importance of clarifying the regulation of cisgenic plants, we argue that this debate is currently too narrowly focused and fails to recognize the broader issues that are persistently addressed within society about GM approaches in general and that, therefore, it is important to broaden the scope of the debate on cisgenic plants. Although the importance to proactively engage in ‘post-research’ issues by researchers involved in the development of LBR GM potato is emphasized and arguably contributes to the recognition of broader concerns, these are often phrased in the sense of ‘educating’ or ‘informing’ public, rather than listening and acting upon their concerns (e.g. [71]). By discussing concerns around the (technical) solution offered, the problem phrasing of the late blight potato disease, the durability of this solution, and patenting and ownership, we hope to contribute to a recognition of the complex socio-ecological, legal and political dimensions in which this technological development is entangled and stimulate discussion that takes this broader view into account. Importantly, we recognize that more concerns can be identified related to the development of cisgenic plants and that we touch upon questions and concerns that would benefit from deeper reflection and further investigation. While concerns mentioned in this paper are specifically formulated around the case of LBR GM potato, we do not consider them to be exclusive to this particular technological development. Rather, we believe that these issues can be found across different agricultural biotechnological developments and approaches—such as CRISPR/Cas9 and synthetic biology—and are therefore important concerns to reflect upon and respond to. Thus, in order to develop truly responsible governance for agricultural biotechnology, the scope of the debate on cisgenic approaches in Europe should be broadened to include other significant concerns raised by stakeholders within the agricultural sector, and the public in general.

## Abbreviations

GM: genetic modification
GMO: genetically modified organism
LBR: late blight resistance
PBR: plant breeders’ right

## Footnotes

1 Several methods to this have been developed of which the Problem Formulation and Option Assessment (PFOA method) developed by Nelson et al. [56] is one. This method is used during the workshops. For more information about this method and the results, see Gillund et al. [38].

2 For clarification, in ‘plant breeding rights’, it is *the variety* that will be the protected property, whereas in patenting it is *the trait*, not the variety, that can be patented.

3 The number of 70–75% is based on personal communication with the stakeholders. As some extra information, the total area of land used for potato production in Norway was 11,835 ha in 2015 as stated by the Norwegian Agency for Agriculture (http://statistikk.landbruksdirektoratet.no/skf/PT912/151610/PT912F21.HTM). Based on the 2500 kg of seed potato on each hectare, the total demand is around 29,587.5 tons of seed potato. However, approximately 10,000 tons of certified seed potato in 2015 was sold, indicating about 34% of the total demand for seed potato.
